# The Role of Brain-Derived Neurotrophic Factor (BDNF) in Depression and Cardiovascular Disease: A Systematic Review

**DOI:** 10.3390/life13101967

**Published:** 2023-09-26

**Authors:** Massimo Fioranelli, Maria Grazia Roccia, Bianca Przybylek, Maria Luisa Garo

**Affiliations:** 1 Department of Human Sciences, Guglielmo Marconi University, Via Plinio 44, 00193 Rome, Italy; m.fioranelli@unimarconi.it (M.F.); m.roccia@unimarconi.it (M.G.R.); 2 Istituto Terapie Sistemiche Integrate, Casa di Cura Villa del Rosario, Via Flaminia 449, 00181 Rome, Italy; bianca.przybylek@goglemail.com; 3 Istituto Terapie Sistemiche Integrate, Casa di Cura Sanatrix, Via di Trasone, 6, 00199 Rome, Italy

**Keywords:** BDNF, brain-derived neurotrophic factor, cardiovascular disease, depression

## Abstract

Background: Several studies have been conducted to prove the bidirectional relationship between cardiovascular disease (CVD) and depression. These two major illnesses share several common risk factors such that the development of either condition may increase the risk of the occurrence of the other. Brain-derived neurotrophic factor (BDNF) has been suggested as a reliable biomarker for depression and a strong predictor of CVD because it plays an important role in neuron survival and growth, serves as a neurotransmitter modulator, and promotes neuronal plasticity. The aim of this systematic review was to examine the bidirectional relationship between CVD and depression, focusing on the potential role of low serum BDNF levels in the development of either disease in the presence of the other. Methods: A systematic search strategy was developed using PRISMA guidelines. Results: Six studies (comprising 1251 patients) were identified, all of which examined the association between CVD and depression. Conclusions: It was found that there may be a strong association between low serum BDNF levels and the risk of post-stroke depression. However, the studies on the role of altered serum BDNF levels and other types of CVD are few. Therefore, the inverse association between depression and CVD cannot be proven.

## 1. Introduction

Cardiovascular disease (CVD) and depression are considered two of the major illnesses occurring worldwide. According to recent statistics, about 523 million people develop CVD each year, and the annual mortality rate for CVD is about 32% (about 19 million deaths per year) [1]. Similarly, depression has an incidence rate of 3.8% (prevalent in 280 million people worldwide) [2]. Both illnesses significantly impact the overall quality of the life of patients and the healthcare system economically due to the associated higher medical costs and the utilisation of health-care services and significantly impact the productive life years of the patients [3,4,5,6,7,8,9].

The large number of studies conducted so far have demonstrated a causal bidirectional relationship between CVD and depression in terms of epidemiological data [10], common risk factors [11], and some specific biological mechanisms [12]. Epidemiological data on the co-occurrence of CVD and depression confirm this close bidirectional relationship and show that a patient has a higher risk of developing one of the two diseases when the other is present. Specifically, patients with depression have a 1.5-fold higher risk of developing CVD than patients without depression [12]. The prevalence of CVD in patients with major depressive disorder has been estimated to be 9.9% (95% CI: 7.4–13.3), with the cardiovascular-related mortality rate being higher than that in non-depressed patients [13]. Conversely, several studies have shown a higher prevalence of major depression in patients with CVD [14]: Major depression or an increase in depressive symptoms affects 20–45% of patients with coronary artery disease [15] and 30–40% of patients with stroke [16,17,18].

Many behavioural and biological mechanisms have been studied to evaluate the determinants of this bidirectional causality, which is likely due to a common pathophysiological pathway [19]: Many common risk factors for both diseases have been identified [12,20]; however, much uncertainty still remains [21]. In recent years, brain-derived neurotrophic factor (BDNF), a neurotrophic growth factor, has been shown to be associated with several neuropsychiatric disorders and some CVDs, such as acute coronary syndrome (ACS) and stroke [22,23,24]; therefore, serum BDNF levels have been proposed as a reliable biomarker for depression [25,26] and a consistent parameter for predicting cardiovascular risk [27,28].

Indeed, according to the neurotrophic hypothesis of depression, depressive symptoms are due to a stress-induced decrease in BDNF [29], because of a mechanism that impairs neuroplasticity and neurogenesis and promotes cell atrophy [30], which is why decreased BDNF levels are often observed in depressed patients [31]. A more complex mechanism links BDNF and CVD: BDNF is involved in angiogenesis and promotes the survival of vascular smooth muscle cells, cardiomyocytes, endothelial cells [32], and atherosclerotic vessels. It also induces oxidative stress by activating the enzyme oxidase in coronary artery smooth muscle cells, which could lead to atherosclerotic plaque instability [33]. High BDNF levels play a protective role against CVD and CVD-related mortality, whereas low serum BDFN levels are considered a risk factor for future coronary events [28,30] and are associated with an increase in risk of future coronary events and mortality in patients with angina [32]. In line with these mechanisms of action, recent evidence has indicated that restoring normal BDNF levels through targeted therapeutic approaches reduces symptomatology in depressed patients [34] and patients with CVD [35].

However, these disease-specific mechanisms of action do not explain the bidirectional relationship. To this end, some hypotheses have been put forward, which are aimed at understanding the biological mechanism underlying the relationship between depression and CVD and thus the co-occurrence of both diseases. Hashimoto (2013) hypothesised that the bidirectional relationship may be due to endoplasmic reticulum (ER) stress, which is of interest in the pathophysiology of both diseases: Under ER stress, unfolded protein response inhibits BDNF secretion through the action of the ER molecular chaperone sigma-1 receptor, which precisely regulates BDNF secretion [19]. Bahls et al. (2019) speculated that the bidirectional relationship could be explained by the fact that BDNF influences both diseases via inflammation, a common feature of depression and CVD [27]. In this case, low BDNF levels in both diseases could be because inflammation tends to decrease BDNF [36].

Although there is evidence to support the hypothesis that alteration of BDNF levels may increase the risk of depression or CVD, the possible role of altered BDNF levels in increasing the risk of depression in patients with CVD and, conversely, the risk of CDV in patients with depression is still unknown. To the best of our knowledge, there are no systematic reviews that have examined a possible bidirectional relationship mediated by BDNF levels. Therefore, the aim of this systematic review is to evaluate the role altered BDNF levels play in patients with CVD and those with depression in terms of increase in risk of depressive symptoms and that of CVD, respectively.

## 2. Materials and Methods

A systematic review of published data was carried out according to the PRISMA guidelines [37].

### 2.1. Eligibility Criteria

Published studies (i.e., peer-reviewed journal articles) that included adult subjects (age ≥ 18 years) with CVD (as defined by the WHO: diseases of the heart and blood vessels, including coronary artery disease, cerebrovascular disease, rheumatic heart disease, and other conditions) and with depressive symptoms (as defined by the Diagnostic and Statistical Manual of Mental Disorders III, IV, or V) diagnosed and confirmed according to the accepted criteria (e.g., TOAST or STEPS-STROKE criteria for stroke, Framingham criteria for heart failure, or Hamilton Depression Rating Scale or Patient Health Questionnaire for depression), which assessed serum BDNF levels on patient admission to the hospital or during follow-up and prospectively investigated a possible association between CVD and depression or vice versa, were included in this study. Studies that analysed BDNF levels in depressed patients and in patients with CVD without examining a possible cause-and-effect relationship, such as case-control studies with healthy subjects or cross-sectional studies, were excluded. Studies that included only diabetic subjects, to rule out a possible interaction between diabetes and CVD, and studies that analysed depressive symptoms in association with other psychiatric symptoms such as anxiety, psychosis, and mixed features, were also excluded. Finally, to reduce the risk of very large heterogeneity amongst the potentially included studies because of different BDNF measurements, studies that investigated BDNF gene polymorphisms or assessed the effect of rehabilitation on BDNF levels were also excluded.

### 2.2. Study Design

Observational studies that comprised patients with CVD and patients with depression and subsequently examined in them the development of depressive symptoms and CVD, respectively, were included.

### 2.3. BDNF Level Measurement

Ninety percent or more of BDNF in the blood is stored in platelets, and there is a close relationship between the platelet count and the serum BDNF concentration. The plasma component of BDNF is believed to be related to particular inflammatory states and the release of specific cytokines, making it relevant and useful as a disease state marker [38]. However, considering that BDNF in platelets may play a role in tissue trauma or nerve injury, BDNF concentrations in serum largely reflect the activation-dependent release of BDNF from platelets, a fact not so easily found in plasma, whose BDNF concentrations during the coagulation process are 200-fold lower than those of serum BDNF, are highly unstable, may reflect the influence of different cellular sources of plasma BDNF, have lower repeatability, and may be influenced by confounding factors such as the patient’s sex and age [38]. Therefore, to analyse the bidirectional relationship, the serum BDNF levels recorded at the time of the diagnosis in patients with either CVD or depression were considered. If serum BDNF levels were also monitored during follow-up, their data were also registered.

### 2.4. Search Strategy

A systematic search strategy was conducted on the three main databases, PubMed, Web of Science, and Scopus, from April to June 2023 without time and language restrictions. The following search strategy was applied: (Brain-derived neurotrophic factor OR BDNF) AND (depression OR depress*) AND (cardiovascular disease). The selection of articles was performed in two steps: In the first step, articles were evaluated by title and abstract, and in the second step, articles that appeared to be suitable for inclusion in the previous step were retrieved and evaluated by reading full texts. Both steps were executed by two independent reviewers (M.G.R. and B.P.). Disagreements between the two reviewers were resolved under the supervision of a third experienced reviewer (M.F.).

The complete list of articles obtained through the systematic search was uploaded on Rayyan (https://www.rayyan.ai). After removing duplicates, potential articles were screened by title and abstract. Post screening, eligible articles that met the inclusion and exclusion criteria were identified and their full texts were retrieved. After that, the two reviewers read the full texts and assessed the eligibility of each study; then, the third and fourth reviewers (M.F. and M.L.G.) checked all the included and excluded studies by reading the full texts and resolved the disagreements between the reviewers. The reasons for exclusion of each article were recorded.

### 2.5. Data Extraction

Data from the included studies were independently extracted and recorded in a datasheet (Excel file) by two authors (M.G.R. and B.P.). No disagreement was registered during this phase. The data that were gathered included (1) study characteristics, i.e., name of the first author, year, country, study design, observation period, enrolled patients’ characteristics (patients with CVD or patients with depression), relationship direction (impact of CVD on development of depression or of depression on that of CVD), patients’ inclusion and exclusion criteria, endpoints, sample size, patients’ age, and sex; (2) diagnostic tools for depression or CVD; and (3) serum BDNF levels. No information was extracted from the figures or graphs reported in the articles.

### 2.6. Quality Assessment

Assessment for bias was performed through the Quality Assessment Tool for Observational Cohort and Cross-Sectional Studies predisposed by the NIH (https://www.nhlbi.nih.gov/health-topics/study-quality-assessment-tools, accessed on 25 June 2023). The tool was composed of 14 items that investigated the quality of the research question, the study population, the methodological aspects (e.g., sample size justification, outcome measurement, and statistical analysis methods), the presence of an adequate timeframe between exposure and outcome, the blinding of outcome assessors, and the follow-up rate. For each item, three possible responses were available: yes, no, or other (CD: cannot be determined, NA: not applicable, NR: not reported). A final rating score was determined for each article, assigning values of 1 for “yes”, 0 for “no”, and −1 for “CD”. The quality was rated as poor if the final score ranged from 0 to 4, fair if the final score ranged from 5 to 10, or good if the final score ranged from 11 to 14.

### 2.7. Data Synthesis

The results were presented as a narrative summary in which the characteristics of the studies were reported in detail.

## 3. Results

### 3.1. Search Results

**Figure 1:**
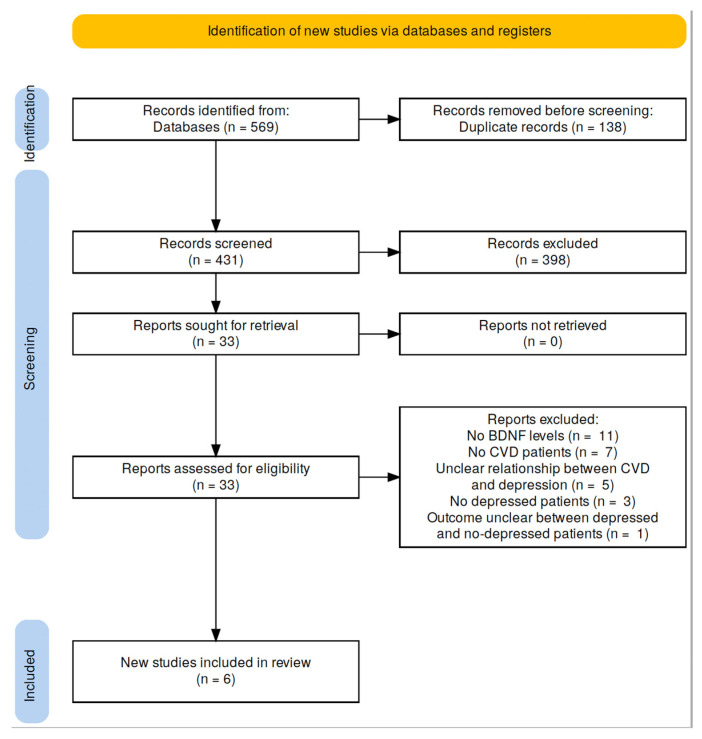
PRISMA flowchart [44].

The search strategy resulted in the collection of 569 articles from the databases: PubMed: 233; Web of Science: 56; Scopus: 233 (Figure 1). After excluding duplicates (n = 138) and screening the remaining (n = 431) articles by reading the title and abstract, 33 articles met the inclusion criteria and were screened by reading the full text. Twenty-seven articles were excluded by reading the full text: 11 studies did not examine BDNF serum levels; 7 and 3 studies did not include patients with CVD and those with depression, respectively; the association between CVD and depression was unclear in 5 studies; and 1 study did not clearly report BDNF serum levels in depressed and nondepressed patients. Finally, six studies published between 2010 and 2022 (n = 1251 patients) were included in the study [30,39,40,41,42,43]. The average age of the patients was approximately 60 years.

### 3.2. Study Characteristics

Four of the six studies were conducted in China [40,41,42,43], one in Germany [30], and one in Brazil [39]. Five were prospective studies, and only one was a retrospective study. Only one was a multicentred study [41]. The observation period was from June 2007 to August 2019, and all of the included studies analysed the relationship between CVD (stroke or coronary artery disease) and the development or worsening of depressive symptoms. Studies that analysed the inverse relationship (role of altered BDNF in the development of CVD in depressed patients) were not included. Five studies investigated possible altered BDNF levels in patients with stroke [39,40,41,42,43]. Only one study examined the role of possible altered serum BDNF levels in the development of depression in patients with coronary heart disease [30]. Stroke was diagnosed using the criteria of STEPS-STROKE [39,43] and TOAST [40,42]. A study that examined serum BDNF levels in patients with minor stroke did not report the criteria for stroke diagnosis [41]. The study, which included patients with coronary artery disease, recruited well-confirmed coronary heart disease patients as determined by medical records [30]. Depressive symptoms were diagnosed with the 9-item Patient Health Questionnaire (PHQ-9), the 17-item Hamilton Rating Scale for Depression (HAMD), and the Montgomery–Åsberg Depression Scale (MADRS). Serum BDNF levels were assessed during enrolment in all of the included studies and monitored during follow-up in only one study. A full description of the characteristics of the included studies is reported in Table 1.

Three of the six studies reported the time of clotting and platelet activation (between 30 and 60 minutes) and the associated temperature (i.e., room temperature or a fixed temperature of 48 °C) for separating the serum [30,42,43]. Serum BDNF levels were measured by highly sensitive and specific fluorometric two-site enzyme-linked immunosorbent assay (ELISA) in all studies: Three studies used a kit to detect mature BDNF [42,43], one study explicitly stated that mature BDNF cannot be distinguished from proBDNF [30], two studies provided no information other than the name of the ELISA manufacturer [39,40], and one study provided no information at all [41]. A full description of the characteristics of the included studies is reported in Table 2.

### 3.3. Risk of Bias

All six studies were rated to be of fair quality. All of the studies did not provide information on investigator blinding and sample size. Only one study analysed BDNF more than once over time [42]. Three of the six studies did not consider potential confounding variables for assessing the relationship between BDNF serum levels and depression. The details of the quality assessment are given in Supplementary Materials Table S1.

### 3.4. Role of BDNF in Patients with Stroke

Five studies comprising a total of 1111 patients (men: 693, women: 418) investigated the possible altered BDNF serum levels in stroke patients (Table 3). Three hundred thirteen patients reported post-stroke depression (PSD) within a maximum of 3 months after stroke onset. One study reported PSD within 14 days of stroke [42].

In the retrospective study by Yang et al. (2010), which prospectively investigated whether serum BDNF is associated with the development of depression in the acute phase, it was found that the mean serum BDNF level in PSD patients was significantly lower than in non-PSD patients as early as 1 day after stroke and that the serum BDNF level < 5.86 ng/mL at day 1 was independently associated with the occurrence of PSD in the acute phase of stroke (OR.28.99; 95% CI, 8.014–104.891; *p* < 0.001 after adjustment) [42].

In 2011, Zhou et al. found that the serum BDNF levels in PSD patients at the time of PSD diagnosis were lower compared with those in stroke patients without PSD in a prospective study of a sample of 112 ischemic stroke patients, 35 of whom had PSD (*p* = 0.027). In the acute phase, no statistically significant differences were observed in the serum BDNF levels between the groups (PSD: 29.1 ± 11.4 ng/mL, no PSD: 28.1 ± 9.7 ng/mL), whereas at follow-up, a statistically significant difference was observed between the two groups (PSD: 21.7 ± 10.3 ng/mL, no PSD: 28.6 ± 9.6 ng/mL, *p* = 0.01). In PSD patients, a significant decrease in BDNF levels was observed from the acute phase till follow-up (acute phase: 29.1 ± 11.4 ng/mL, follow-up: 21.7 ± 10.3 ng/mL, *p*-value not reported in the primary study). On comparison, no changes in the BDNF serum levels were seen in patients without PSD between 7 days and 6 months after the stroke [43].

In the study by Li et al. (2018), which was conducted on a sample of 216 patients with a first episode of acute ischemic stroke diagnosed via TOAST and composed of a higher number of women (women: 128, men: 88), the authors reported an incidence of major depression of 27.3%. The BDNF serum level was significantly lower in depressed patients (median: 8.1 ng/mL, IQR: 5.6–9.4) than in non-depressed patients (median: 13.7 ng/mL, IQR: 10.4–16.5, *p* < 0.0001). Moreover, the authors demonstrated a significant relationship between lower serum BDNF levels at admission and a higher HAM-D score at 3 months (r = 0.361, *p* < 0.0001), even in the adjusted model (β = 0.304, *p* = 0.009) [40].

Baccaro et al. (2018) demonstrated in a preliminary Brazilian prospective study aimed to evaluate the role of multifactorial alterations, including poststroke anatomic lesions and biomarker levels, in the development of PSF and conducted according to strict exclusion criteria that 13.6% of patients, 57.1% of whom had a previous depressive episode before the psychiatric interview using SCID-1, had PSD within 3 months of stroke. No statistically significant serum BDNF levels were seen between patients with and without PSD (*p* = 0.35) [39].

A recent multicentre study by Qiu et al. (2022), which included 530 patients with minor stroke (NIHSS score ≤ 3 points) and excluded patients with a history of depression, anxiety, mental illness, or taking psychotropic drugs, showed an incidence rate of 31.7% of PSD within 3 months of stroke. In a multivariate logistic regression analysis aimed at assessing the probability of PSD occurrence, the authors reported a significant difference in BDNF levels (*p* = 0.029, OR = 0.916, 95% CI: 0.846–0.991) between women with PSD and those without PSD, whereas no such difference was observed in men [41].

### 3.5. Coronary Heart Disease and Altered BDNF Levels

Only one study that investigated the possible role of altered BDNF levels in the development of depression in patients with coronary artery disease was included in this review [30] (Table 4). This nonexperimental prospective study was conducted from December 2012 to November 2014 in a sample of 190 CHD patients, which include 48 patients with persistent depression (patients who showed depressive symptoms at enrolment and during follow-up), 23 patients with incident depression (patients without depressive symptoms at enrolment but who developed depressive symptoms during follow-up), 25 patients with remitted depression (patients who showed depressive symptoms at enrolment that disappeared during follow-up), and 94 patients without any form of depression (patients without depressive symptoms throughout the study period). The analysis of BDNF levels showed a statistically significant difference between the four depressed groups (*p* = 0.002). Post-hoc comparisons revealed that the BDNF levels were lower in permanently depressed patients compared with those in non-depressed patients and those in patients with incident depression (*p*-values < 0.05), even in the adjusted model. The BDNF levels in permanently nondepressed patients did not differ significantly from those in patients who remitted during follow-up (*p* = 0.643) or from those in patients with acute depression (*p* = 0.371). In initially nondepressed patients, lower BDNF levels were not associated with the onset of depression (OR, 1.50; 95% CI, 0.95–2.39; *p* = 0.081), whereas in patients who were depressed at diagnosis, serum BDNF levels were predictive of depressive symptoms at 6 months (OR, 0.37; 95% CI, 0.19–0.74; *p* = 0.005). In addition, in the case of patients with acute coronary syndrome, the risk of depression was higher in patients with lower BDNF serum levels (OR, 4.60; 95% CI, 1.12–18.97; *p* = 0.035).

## 4. Discussion

CVD and depression are major illnesses that are interrelated. Several clinical studies have already demonstrated a poor medical prognosis for patients suffering from both conditions [45,46]. A kind of dose–response relationship has been proposed between the severity of depression and cardiac prognosis in depressed patients [47,48], and conversely, an increase in depressive symptoms has been observed in patients with CVD [46,49]. Investigating the biological mechanisms underlining this bidirectional relationship, some animal and human studies have hypothesised that BDNF might play a mediating role in the onset of one of these two diseases because lower serum BDNF levels were observed in patients of both CVD and depression [50,51,52,53]. Nevertheless, the role that serum BDNF levels plays in the development of either disease in the presence of the other seems unclear. The aim of this systematic review was to investigate this bidirectional relationship mediated by serum BDNF levels and to explain whether and to what extent altered serum BDNF levels might serve as biomarkers for predicting the development of depression and CVD in patients diagnosed with CVD and patients diagnosed with depression, respectively.

### 4.1. Relationship between Stroke and Depression

The findings of this study have provided evidence of a clear association between decreased BDNF serum levels and the subsequent development of depressive symptoms in stroke patients: Patients who had low serum BDNF levels at the time of stroke diagnosis had a higher risk of developing depression in the PSD period. To understand the underlying mechanism of this relationship mediated by the serum BDNF levels, it is necessary to understand that the BDNF can cross the blood-–brain barrier in both directions [54] and thus consider the changes in BDNF serum levels as a reflection of the changes occurring in the BDNF levels in the brain. Following this assumption, the lower serum BDNF levels in PSD could indicate a breakdown of the stress adaptation system and its failure to protect the brain from stress-induced neuronal degeneration [42], which consequently sets the stage for the development of depression [55]. The mechanism of the action of serum BDNF in the development of depression could be explained by the BDNF changes that occur in the brain immediately after stroke. Under normal conditions, the brain’s adaptive response to stroke is to increase BDNF levels to reduce neuronal loss and promote subsequent neurogenesis. However, in some patients, this adaptive response does not occur, and a decrease in BDNF is observed, likely due to a downstream induction of BDNF, which is the result of altered neuronal excitability with a downstream signal of excitatory neurotransmitters [56]. The consequence of this BDNF decline is neuronal loss and a slowing of the neurogenesis processes, which in turn are possible mechanisms in the pathogenesis of depression [57]. This mismatch is confirmed by the analysis of the correlation between BDNF decline and stroke severity: All of the included studies reported significantly higher NIHSS scores in patients who had lower BDNF levels at diagnosis and reported depression in the post-stroke period [40,42,43]. Serum BDNF levels are therefore a signal of maladaptation or dysfunction of the body leading to stroke and could therefore be used as a potential biomarker for PSD. Restoring BDNF levels in post-stroke patients has been proposed as a reliable method to reduce the risk of the development of PSD in them [24].

### 4.2. Relationship between Coronary Artery Disease and Depression

On examining the role of BDNF in the development of depression after coronary artery disease (ACS), a different role of BDNF was identified in this study. According to the only study included in our work that examined the relationship between CVD distinct from stroke and the occurrence of depression after coronary artery disease [30], the role of lower serum BDNF levels is rather uncertain. Although lower BDNF levels during follow-up after ACS were observed in patients with depression, no cause–effect relationship was established between lower serum BDNF levels on the diagnosis of ACS and the subsequent development of depression. In other words, although lower serum BDNF levels on ACS diagnosis were predictors of the persistence of depression in those patients who had depression before the ACS event, they did not occur in patients with incidence of depression (i.e., depression developing during the follow-up period after ACS). In patients with incidence of depression, its development during the follow-up period appears to be related to the severity of CVD and is associated with more severe treatments and longer hospital stays [30] but not with low serum BDNF levels. Thus, in the presence of concomitant CVD and depression at the time of ACS diagnosis, the role of low BDNF levels remains unclear; therefore, we cannot assess whether these lower levels represent a state of depression or a marker of it. According to the study by Bus et al. (2011), BDNF levels in depressed patients decrease over time, suggesting that the likelihood of a patient persisting in a depressed state increases once biological changes take place [58]. The role that CVD plays in exacerbating depressive symptomatology can certainly be suspected, but a possible role of BDNF in this process seems less clear.

BDNF and its receptors (tyrosine receptor kinase B) are expressed in the peripheral vessel wall, where it stimulates angiogenesis, promotes endothelial cell survival, favours neovascularisation in response to hypoxic stimuli via the Akt signalling pathway, maintains vascular integrity [59], and plays a role in cardiomyocyte contractility [60,61] and activation of enzymes involved in the detoxification of reactive oxygen species [62]. Low BDNF levels have been frequently observed in patients with coronary artery disease and heart failure (HF) and have been associated with HF severity and unfavourable outcome in HF patients, whereas higher serum BDNF levels have been associated with a lower risk of CVD and mortality [63]. On analysing the post-myocardial infarction (MI) phase, animal studies have shown that BDNF plasma levels are significantly higher in mice with myocardial ischemia, and BDNF expression is upregulated by neural signals from the heart after MI to protect the myocardium from ischemic damage: BDNF/TrkB indeed attenuates ischemic heart damage and inhibits cardiomyocyte apoptosis by regulating TRPC3/6 channels [60,64,65]. The same mechanism has been demonstrated in patients with unstable angina, in whom significantly higher levels of BDNF were observed in the coronary sinus and aorta in response to the cardiac event, whereas no such signs were observed in patients with stable angina and in patients without coronary artery disease [33]. Thus, if, on the one hand, it is clear that BDNF contributes to the pathophysiology of coronary artery disease and has therefore been proposed as a potential diagnostic tool [66], an elevated BDNF level may, on the other hand, represent a compensatory response to the heart and vessels on injury [67].

This mechanism may explain the lack of evidence in the findings of this study for a possible role of BDNF in developing depression after coronary artery disease. Studies examining the role of BDNF in the relationship between CVD (other than stroke) and depression are needed along with those investigating the BDNF response to cardiac events. However, the findings of this study have their limitations: It is unclear whether the eligibility criteria of this study resulted in high specificity or whether the studies that investigated this specific relationship are less likely to be conducted or published because of nonsignificant results. 

### 4.3. Relationship between Depression and CVD

There was a lack of studies on the analysis of the other side of the bidirectional relationship, that is, between depression and CVD, and therefore, not a single study was included in this review. From the cross-sectional studies reported in this review, it appears that there is a possible association between low BDNF levels and depression and CVD as concurrent conditions [32], but prospective studies are needed to determine a possible cause–effect relationship.

### 4.4. Limitations of the Included Studies

However, the studies that were included in this review had some methodological limitations. First, significant heterogeneity was found in the included studies regarding the procedures used to determine BDNF serum levels in terms of centrifugation strategy, temperature during clotting, and choice of bioassay. The major concerns were related to clotting time and detection of mature BDNF. Only three studies reported the clotting time, which ranged from 30 to 120 minutes, with the temperature varying from room temperature to 48 °C. Only two of these three studies used a kit for a selective detection of mature BDNF. The consequences of this level of uncertainty may have significantly affected the accuracy of the analytical procedure and led to bias in the BDNF results. According to this study, which suggests a clotting time of 1 hour given the rapid release of serum BDNF from platelets during clotting at room temperature within the first hour after sample collection [54] and the use of ELISA kits capable of selective discrimination between mature BDNF and proBDNF [68,69], future studies should use not only more standardised procedures to allow for meta-analytic comparisons amongst studies but also accurate analytical methods that allow for reliable measurements of serum BDNF levels. Second, serum BDNF levels are influenced by platelets, and some studies have demonstrated an association amongst depression, CVD, and platelet function [38,70]. None of the included studies examined the relationship between BDNF and platelet reactivity. Future studies should examine the ratio of pro-BDNF to mature BDNF, as suggested by some authors [71,72], and also apply a proteomic approach that could expand the understanding of BDNF pathways [73]. Finally, BDNF, depression, and CVD could be studied together with inflammatory parameters related to depressive symptoms [74].

### 4.5. Limitations of this Systematic Review

This work has some limitations. First, the number of included studies was very small because strict eligibility criteria were applied, which, on the one hand, increased the specificity of this work but on the other, significantly limited its potential. The a priori decision to exclude an analysis of studies that investigated a BDNF polymorphism such as Val66met and BDNF methylation certainly had an impact on the number of included studies. Considering the aim of this work, a more in-depth analysis also examining genetic variants such as Val66met could have introduced a relevant heterogeneity factor that would have shifted the focus of this study. Although low serum BDNF levels are common in both diseases, the role of polymorphisms in some cardiovascular events is still unclear [75]. Thus, during the analysis of the role of BDNF in the incidence/prevalence of either disease in the coexistence of the other, the unclear role of BDNF polymorphisms may have hindered the investigation of the bidirectional relationship in this study. BDNF methylation was excluded because the role of this parameter as a suitable biomarker for psychiatric disorders is not well understood [76]. Second, due to the large heterogeneity of studies, a meta-analysis could not be performed, although quantitative data on BDNF serum levels were available, which would have favoured a quantitative approach. 

## 5. Conclusions

Serum BDNF levels have been shown to be a valuable biomarker for predicting PSD: indeed, low serum BDNF levels are strongly associated with a higher risk of the development of PSD in patients. The role of BDNF in the development of depression in patients after cardiovascular events other than stroke should be investigated, including the assessment of BDNF alteration in response to cardiovascular injury. Further studies are also needed to understand whether and to what extent low serum BDNF levels can increase the risk of the development of CVD in depressed patients. 

In conclusion, from the findings in this study it is evident that BDNF plays an important role in explaining one side of the bidirectional relationship between CVD and depression; therefore, serum BDNF level could be considered as a valuable biomarker. In the clinical context, restoration of BDNF levels could therefore be a novel therapeutic target to prevent or reduce depressive symptoms in patients with CVD.

## Figures and Tables

**Table 1:** Study characteristics.

Author	Country	Study Design	Observation Period	Relationship Direction	CVD at Recruitment	CVD Diagnosis	Outcome	Outcome Evaluation	Endpoints
Baccaro et al. (2019) [39]	Brazil	Prospective	April 2006–September 2014	CVD → depression	Stroke	STEPS-STROKE criteria confirmed by CT + TOAST for ischemic stroke	PSD	PHQ-9 ≥ 10	Within 3 months from stroke onset (subclinical phase)
Kuhlmann et al. (2017) [30]	Germany	Prospective	December 2012–November 2014	CVD → depression	CHD	Medical charts	Depressive symptoms	PHQ-9 ≥ 7	PHQ-9 at baseline and at 6 months
Li et al. (2014) [40]	China	Prospective	NR	CVD → depression	First episode of acute ischemic stroke	TOAST	Depression	HAM-D	Baseline and after 3 months from stroke
Qiu et al. (2022) [41]	China	Prospective	May 2018–August 2019	CVD → depression	Minor stroke (NIHSS score < 3)	Confirmed by CT or MRI	PSD	HAM-D > 7	Baseline and after 3 months from stroke
Yang et al. (2011) [42]	China	Prospective	June 2007–June 2008	CVD → depression	Stroke with acute cerebral infarction	TOAST	PSD	HAM-D and MADRS	3–7–14 days after admission
Zhou et al. (2011) [43]	China	Retrospective	April 2008–November 2008	CVD → depression	Ischemic stroke	WHO criteria	PSD	HAM-D	7 days and at 1, 3, and 6 months after onset of stroke

BDNF: brain-derived neurotrophic factor; CHD: coronary artery disease; CT: computed tomography, CVD: cardiovascular disease; MRI: magnetic resonance imaging; HAM-D: Hamilton Rating Scale for Depression; PHQ: Patients Health Questionnaire; TOAST: Trial of Org 10,172 in Acute Stroke Treatment; WHO: World Health Organization.

**Table 2:** Analytical methods for BDNF determination.

Author	Analytical Methods	BDNF Evaluation	Differentiation mBDNF from proBDNF	Endpoints	Sample Size
Baccaro et al. (2019) [39]	(1) Sample collection (2) Aliquots frozen at −80 °C	ELISA (HMYOMAG-56k-02, Millipore^®^, St. Charles, MO, USA)	Unclear	Within 3 months from stroke onset (subclinical phase)	103
Kuhlmann et al. (2017) [30]	(1) Sample collection (2) Clotting time 30–60 min at room temperature (3) Centrifugation at 3.500 rpm for 15 min at 4 °C (4) Serum refrigeration at 20 °C	ELISA (Promega Inc., Mannheim, Germany)	No	PHQ-9 at baseline and at 6 months	190
Li et al. (2014) [40]	(1) Sample collection (2) Samples stored before analysis at −80 °C	ELISA (DuoSet ELISA Development, R&S Systems, USA)	Unclear	Baseline and after 3 months from stroke	216
Qiu et al. (2022) [41]	NR	NR	NR	Baseline and after 3 months from stroke	530
Yang et al. (2011) [42]	(1) Sample collection (2) Kept at room temperature for 1 h (3) Kept for 1 h at 48 °C (4) Centrifugation at 2000× *g* for 10 min at 48 °C (5) Kept frozen at −80 °C	ELISA (Promega Inc., Madison, WI, USA)	Yes	3–7–14 days after admission	100
Zhou et al. (2011) [43]	(1) Sample collection (2) Kept at room temperature for 30 min (3) Centrifugation for 15 min at 1000× *g* (4) Stored at −80 °C	BDNF Emax Immunoassay System kit (R&D Systems, Minneapolis, MN, USA)	Yes	7 days and at 1, 3, and 6 months after onset of stroke	112

**Table 3:** Relationship between stroke and depression.

Author	Sample Size	Follow-Up	Age	Sex (Male/Female Ratio)	NIHSS	Number of Patients with Depression	Findings
Baccaro et al. (2019) [39]	103	71 days	Median: 63 years	60/43	NR	14	No statistically significant difference in serum BDNF levels between patients with and without PSD (*p* = 0.35)
Li et al. (2014) [40]	216		Depressed patients: 72.8 ± 11.2; not depressed patients: 63.9 ± 9.1	88/128	Depressed: median 8 (IQR 4–14); not depressed: median 5 (IQR 2–8)	59	BDNF serum levels in depressed patients: 8.1 ng/mL (5.6–9.4); BDNF serum level in not depressed patients: 13.7 ng/mL (10.4–16.5), *p* < 0.0001. Inverse correlation between lower serum BDNF levels at admission and higher HAM-D score at 3 months (r = 0.361, *p* < 0.0001).
Qiu et al. (2022) [41]	530		Female: 58.8 ± 12.3; male: 58.0 ± 11.5	415/115	NIHSS < 3	168	Serum BDNF levels statistically different in women with and without PSD: *p* = 0.029, OR = 0.916, 95% CI: 0.846–0.991
Yang et al. (2011) [42]	100		PSD: 68.95 ± 9.28; no PSD: 68.43 ± 11.18; HC: 65.12 ± 10.27	77/73	PSD: median 7 (IQR 4~9.5); no PSD: median 3 (IQR 2~4)	37	Serum BDNF levels lower in PSD patients than in non-PSD patients 1 day after stroke. No significant differences on day 7 (F = 2.796, *p* = 0.064).
Zhou et al. (2011) [43]	112	6 mo.	PSD: 61.7 ± 8.5; no PSD: 63.5 ± 12.5	53/59	PSD: median 7 (IQR 1–24); no PSD: median 5 (IQR 1–13)	35	Diagnosis, serum BDNF level lower in PSD patients compared to non-PSD patients (*p* = 0.027). Acute stage: no significant differences. No significant differences in patients without PSD comparing BDNF levels at 7 days and 6 months

IQR: interquartile range; PSD: post-stroke depression.

**Table 4:** Relationship between cardiovascular events and depression.

Author	Sample Size	Follow-Up	Age	Sex (Male/ Female Ratio)	Number of Patients with Depression	Findings
Kuhlmann et al. (2017) [30]	190		65 ± 11	145/45	Incident depressed: 23; persistently depressed: 48; remitted depressed: 25, persistently non-depressed: 94	Depressed patients: BDNF significantly lower in persistently depressed patients (*p* < 0.05). BDNF not predictive for depression in incident depressed patients (OR: 1.50, 95% CI: 0.95–2.39, *p* = 0.081). Persistent depressive symptoms more common in patients with lowed BDNF concentration at admission (OR: 0.37, 95% CI: 0.19–0.74, *p* = 0.005). Acute coronary syndrome predictive factor for depressive symptoms (OR: 4.60, 95% CI: 1.12–18.97, *p* = 0.035). Charlson Comorbidity Index significantly predicts depressive symptoms in initially non-depressed patients (OR: 1.61, 95% CI: 1.06–2.46, *p* = 0.026).

## Data Availability

Data used in the manuscript are inserted in the main text and tables.

## Supplementary Materials

The following supporting information can be downloaded at: https://www.mdpi.com/article/10.3390/life13101967/s1, Table S1: Risk of bias.
